# Multi-Step Attack Detection Based on Pre-Trained Hidden Markov Models

**DOI:** 10.3390/s22082874

**Published:** 2022-04-08

**Authors:** Xu Zhang, Ting Wu, Qiuhua Zheng, Liang Zhai, Haizhong Hu, Weihao Yin, Yingpei Zeng, Chuanhui Cheng

**Affiliations:** 1 School of Cyberspace Security, Hangzhou Dianzi University, Hangzhou 310018, China; zhangxu@hdu.edu.cn (X.Z.); wuting@hdu.edu.cn (T.W.); zhailiang125@hdu.edu.cn (L.Z.); huh2o2@hdu.edu.cn (H.H.); yinweihao@hdu.edu.cn (W.Y.); yzeng@hdu.edu.cn (Y.Z.); 2 School of Information and Safety Engineering, Zhongnan University of Economics and Law, Wuhan 545001, China; cch@zuel.edu.cn

**Keywords:** multi-step attack detection, Hidden Markov Model, pre-training

## Abstract

Currently, hidden Markov-based multi-step attack detection models are mainly trained using the unsupervised Baum–Welch algorithm. The Baum–Welch algorithm is sensitive to the initial values of model parameters. However, its training uses random or average parameter initialization methods, which frequently results in the model training into a local optimum, thus, making the model unable to fit the alert logs well and thereby reducing the detection effectiveness of the model. To solve this issue, we propose a pre-training method for multi-step attack detection models based on the high semantic similarity of alerts in the same attack phase. The method first clusters the alerts based on their semantic information and pre-classifies the attack phase to which each alert belongs. Then, the distance of the alert vector to each attack stage is converted into the probability of generating alerts in each attack stage, replacing the initial value of Baum–Welch. The effectiveness of the proposed method is evaluated using the DARPA 2000 dataset, DEFCON21 CTF dataset, and ISCXIDS 2012 dataset. The experimental results show that the hidden Markov multi-step attack detection method based on pre-training of the proposed model parameters had higher detection accuracy than the Baum–Welch-based, K-means-based, and transfer learning differential evolution-based hidden Markov multi-step attack detection methods.

## 1. Introduction

With the rapid development of network technology, security has become an issue on the internet. IDSs [1] have been employed to monitor network transmissions instantly according to pre-set rules to detect network attacks promptly. However, attacks often cause the IDS to issue many alerts, which leads to truly high-threat attacks being submerged in these alerts. In this regard, we proposed to dig out the logical relationship between various attacks, namely, multi-step attacks (MSA). The MSA is defined as a combined attack that consists of multiple attack phases. Each phase of MSA consists of a single-step attack, and there is a logical cause-and-effect relationship between the attack phases [2]. Compared with intrusions based on individual packets, MSAs last longer and are more threatening.

Currently, the approaches for MSA detection can be main divided into two categories: similarity-based methods and model matching methods [2,3]. The similarity-based methods detect MSA sequences based on the correlation of alert types and the similarity of IP addresses, ports, etc. Zhu et al. [4] proposed an alert correlation matrix in the calculation of alert similarity to express the alert similarity in terms of the correlation strength of alert types. Wang et al. [5] further constructed an alert attribute similarity weight matrix using the IP address, port, time interval, and alert type of the alert to represent the alert similarity.

Chi-hung et al. [6] proposed a dynamic alert similarity matrix update algorithm based on the original alert similarity, using the original equality constraint set. These methods are fast but have low accuracy. The model matching methods assume that various MSAs take distinct attack phases. These methods first model the attack steps of various MSA types and then determine the most likely MSA sequence based on the detection model. Compared with similarity-based methods, the model matching methods have higher accuracy and a faster detection speed. Supervised and unsupervised are two training methods of model matching.

In the area of MSA detection, the most frequently employed supervised learning models are HMM, LSTM, CNN, and GCN. The HMM MSA detection methods [7,8,9] based on supervised learning can directly compute model parameters using a labeled dataset and then label alert sequences online using the Viterbi algorithm. The LSTM is a more sophisticated version of the HMM. It is more powerful than HMM at labeling sequences but requires more training data [10,11,12].

CNN is a classic supervised deep-learning framework that is primarily used for feature extraction. In MSA detection, a CNN model [13] is often used to eliminate false positive alerts and fuse MSA chains. GCN is used to handle non-Euclidean data that CNN cannot handle, such as MSA graphs [14], while supervised learning is better at detecting MSAs, and they still rely on alert labels. In MSA detection, it is difficult to obtain alerts with labels, and the complete attack process must be checked for alert labeling, which largely relies on expert knowledge.

Unsupervised training, in comparison to supervised training, has the advantage of training the model without expert knowledge; however, the detection effect is typically lower. Bayesian Networks and HMM are the most common unsupervised learning models in the field of MSA detection. Bayesian network-based MSA detection [15,16] builds a Bayesian attack graph to detect MSA and predict the attacker’s next target. The HMM is generally considered as a special Bayesian network that uses the original Baum–Welch algorithm to train the model parameters by fitting alert logs [17,18,19,20,21]. The alert sequence is then decoded by the Viterbi algorithm into the corresponding attack phase sequence, which enables MSA detection.

Although the importance of HMMs based on unsupervised learning in MSA detection has been widely recognized in the field, the following problems still exist:

A It is generally known that the Baum–Welch algorithm is very sensitive to the initialization values. The current Baum–Welch algorithm uses average or random initialization methods to initialize HMM. However, this initialization method can easily lead the multi-step attack detection model into local optimal solutions, which reduces the detection effectiveness of the model.
B The alert description generated by network interaction in the same attack stage has high semantic similarity and can be used to distinguish each attack stage. However, in the current HMM-based MSA detection method, category coding is used to encode alert description attributes, losing the rich semantic information of the alert description.

To solve the above problem, an initial parameter training method for multi-step attack detection models is proposed in this paper based on the concept of high semantic similarity of alerts in the same attack phase. Semantically similar alerts are used to optimize the initial HMM parameters. This method pre-divides the attack stage to which each alert belongs by clustering the semantic information of the alert. Then, we transfer the knowledge learned in the pre-training to the initialization process of the downstream hidden Markov Model to optimize the model parameters, thereby, improving the performance of the model. The main contributions of this paper are as follows.

A We propose a method to cluster the alerts with similar semantics into the same attack phase based on the semantic information of alerts. The method first uses the alert descriptions to train the word embedding model, then uses this trained model to convert the alert descriptions into alert vectors, and finally clusters the alert vectors by K-means.
B For the initial value used in the training of the current Baum–Welch algorithm, it is easy to cause the multi-step attack detection model to fall into the local optimum problem. We propose an initial value optimization method based on alert semantic information. The method argues that the smaller the distance between the alert vector and the center point of the cluster, the higher the probability of that cluster (attack phase) generating an alert. Based on this idea, we first use the method proposed in A for semantic clustering and then convert the distance between each cluster center and the alert vector into the probability of generating alerts in the attack phase, instead of the initial HMM parameters. Finally, we train the model by the Baum–Welch algorithm. In this paper, the initial parameters of the Baum–Welch algorithm are optimized by semantic information, which avoids the problem that the original initial value makes the model fall into a local optimum, thereby, improving the detection effect of the model.
C We evaluated the proposed MSA detection model with the DARPA 2000 dataset, DEFCON21 CTF dataset, and ISCXIDS 2012 datasets. Compared with MSA detection models trained by the Baum–Welch method, K-means method, and transfer-learning-based Different Evolution method, our results show that the MSA detection model trained by the proposed method had a better detection effect.

The remaining sections of this paper are organized as follows. Section 2 reviews relevant concept of HMM. Section 3 describes in detail the proposed pre-training method and the scheme of MSA detection based on HMM. Section 4 performs model evaluation. Finally, Section 5 summarizes our work.

## 2. Preliminaries

The concept used in this paper is the Hidden Markov Model (HMM), which is briefly explained in the following.

### 2.1. Definition

A Hidden Markov Model (HMM) is a Markov process with unobservable parameters. The hidden state cannot be observed directly in this model but can be determined through the sequence of observations. The HMM model is represented by a triplet λ=(A,B,π). In the triplet, *A* denotes the hidden state transfer matrix, *B* denotes the probability matrix generated by the observed state, and π denotes the initial hidden state probability distribution. The HMM makes the following assumptions.

1. Homogeneous Markov chain hypothesis. The hidden state at any time only depends on its previous hidden state.
2. Observational independence assumption. The observed state at any moment only depends on the hidden state at the current moment.

### 2.2. Baum–Welch Algorithm

The Baum–Welch algorithm [22] is a classic unsupervised HMM training algorithm, which uses the principle of the EM algorithm to train parameters λ. Knowing the observed state sequence $O=\{o_{1},o_{2},\cdots ,o_{T}\}$, *o* indicates the observed state at each moment. The unknown sequence of hidden states is denoted by $Q=\{q_{1},q_{2},\cdots ,q_{T}\}$, *q* indicates the hidden state at each moment. The derivation of the procedure for solving the HMM parameters λ is as follows.

<1> For the observed series *O*, the model parameters are solved by the EM [23] iterative formula as shown in Equation (1). *g* indicates the number of current iterations.
  (1) $$\lambda^{\left(g+1\right)}=\arg\max_{\lambda }\int_{Q}\log P\left(O,Q|\lambda \right)P\left(Q|O,\lambda^{\left(g\right)}\right)dQ$$
<2> Since $\lambda^{\left(g\right)}$ is known in the iterative process, $P(O|\lambda^{\left(g\right)})$ can be regarded as a constant and introduced into Equation (1) to convert $P(Q|O,\lambda^{\left(g\right)})$ into a joint distribution to simplify the calculation as shown in Equation (2).
  (2) $$\begin{array}{cc} \lambda^{\left(g+1\right)} & =\arg\max_{\lambda }\int_{Q}\log P\left(O,Q|\lambda \right)P\left(Q|O,\lambda^{\left(g\right)}\right)P\left(O|\lambda^{\left(g\right)}\right)dQ\\ \; & =\arg\max_{\lambda }\int_{Q}\log P\left(O,Q|\lambda \right)P\left(Q,O|\lambda^{\left(g\right)}\right)dQ \end{array}$$
<3> Use the properties of discrete variable probability distribution integrals to expand Equation (2) as shown in Equation (3).
  (3) $$\begin{array}{cc} \lambda^{\left(g+1\right)} & =\arg\max_{\lambda }\sum_{q_{1}=1}^{N}\sum_{q_{2}=1}^{N}\cdots \sum_{q_{T}=1}^{N}\log P\left(O,Q|\lambda \right)P\left(Q,O|\lambda^{\left(g\right)}\right) \end{array}$$
<4> Decompose P(O,Q|λ) as shown in Equation (4). $a_{i,j}$ is an element of matrix *A*, which represents the hidden state transition probability. $b_{i}\left(j\right)$ is an element of matrix *B*, which represents the probability that the hidden state produces the observed state.
  (4) $$\begin{array}{cc} \log P(O,Q|\lambda ) & =\log\pi_{q_{1}}\prod_{t=2}^{T}P\left(q_{t}|q_{t-1}\right)\prod_{t=2}^{T}P\left(o_{t}|q_{t}\right)\\ \; & =\log\pi_{q_{1}}+\sum_{t=2}^{T}\log P\left(q_{t}|q_{t-1}\right)+\sum_{t=2}^{T}\log P\left(o_{t}|q_{t}\right)\\ \; & =\log\pi_{q_{1}}+\sum_{t=2}^{T}\log a_{i,j}+\sum_{t=2}^{T}\log b_{i}\left(j\right) \end{array}$$
<5> Bring Equation (4) into Equation (3), and we find Equation (5).
  (5) $$\begin{array}{cc} \lambda^{\left(g+1\right)}=\arg\max_{\lambda }\sum_{q_{1}=1}^{N}\sum_{q_{2}=1}^{N}\cdots \sum_{q_{T}=1}^{N}[\log\pi_{q_{1}}+\sum_{t=2}^{T}\log a_{i,j}+ & \\ \sum_{t=2}^{T}\log b_{i}\left(j\right)]P\left(Q,O|\lambda^{\left(g\right)}\right) \end{array}$$
<6> Solve for each parameter separately using the Lagrange multiplier method. The results are shown in Equations (6)–(8).
  (6) $$\begin{array}{cc} \pi_{i} & =\frac{P(q_{1}=i,O|\lambda^{\left(g\right)})}{\sum_{i=1}^{N}P\left(q_{1}=i,O|\lambda^{\left(g\right)}\right)} \end{array}$$
  (7) $$\begin{array}{cc} a_{i,j} & =\frac{\sum_{t=1}^{T}P\left(q_{t-1}=i,q_{t}=j,O|\lambda^{\left(g\right)}\right)}{\sum_{t=1}^{T}P\left(q_{t-1}=i,O|\lambda^{\left(g\right)}\right)} \end{array}$$
  (8) $$\begin{array}{cc} b_{i}\left(j\right) & =\frac{\sum_{t=1}^{T}P\left(q_{t}=i,o_{t}=j|\lambda^{\left(g\right)}\right)}{\sum_{j=1}^{M}\sum_{t=1}^{T}P\left(q_{t}=i,o_{t}=j|\lambda^{\left(g\right)}\right)} \end{array}$$

The Baum–Welch algorithm uses the result of the final derivation of the formula to train the HMM parameters until the model converges, where $\lambda^{\left(0\right)}$ uses randomized initial values or average initial values.

## 3. Multi-Step Attack Detection

This section provides a detailed description of the proposed MSA detection scheme. First, a formal representation of the MSA model is given. Then, the training and detection process is described. Next, the pre-training method of the detection model parameters is described in detail. Finally, the online detection method is given.

### 3.1. Multi-Step Attack Detection Model Definitions

This section gives the HMM for MSA detection, represented as a six-tuple <S,V,O,A,B,π>. The specific definitions of each parameter in the six-tuple are shown in Equations (9)–(17).

<1> Set of attack phases *S*. It consists of *N* phases of an MSA.
  (9) $$S=\{s_{1},s_{2},\cdots ,s_{N}\}$$
<2> Alert description set *V*. It represents the set of *M* possible alert description generated by the IDSs.
  (10) $$V=\{v_{1},v_{2},\cdots ,v_{M}\}$$
<3> Alert description sequence *O*. It is an alert description sequence of length *T* generated by IDSs.
  (11) $$O=\{o_{1},o_{2},\cdots ,o_{T}\}$$
<4> The attack phase transition matrix *A*. It represents the probability that phase *i* at the moment t−1 convert to phase *j* at the moment *t* in the set of MSA phases *S*.
  (12) $$a_{ij}=p\left(q_{t}=s_{j}|q_{t-1}=s_{i}\right)$$
  (13) $$A=\left[\begin{array}{ccc} a_{11} & \cdots & a_{1N}\\ \vdots & \ddots & \vdots \\ a_{N1} & \cdots & a_{NN} \end{array}\right]$$
<5> The alert description transition probability matrix *B*. It represents the probability that alert description $v_{j}$ is generated at the moment *t* by the MSA phase *i*.
  (14) $$b_{i}\left(j\right)=p\left(o_{t}=v_{j}|q_{t}=s_{i}\right)$$
  (15) $$B=\left[\begin{array}{ccc} b_{1}\left(1\right) & \cdots & b_{1}\left(M\right)\\ \vdots & \ddots & \vdots \\ b_{N}\left(1\right) & \cdots & b_{N}\left(M\right) \end{array}\right]$$
<6> The initial attack phase probability matrix π. It represents the probability of each attack phase at the initial moment t=1.
  (16) $$\pi =p(q_{1}=s_{i})$$
  (17) $$\pi =\{\pi_{1},\pi_{2},\cdots ,\pi_{N}\}$$

The above model maps the alert sequence generated by the IDS to the observed sequence of the HMM and the sequence of MSA phases to the hidden state sequence of the HMM. Based on this HMM, the MSA detection can be transformed into a task that finds the sequence of attack phases that are most likely to generate the alert description sequence *O*. It is necessary to solve this task through the following steps: (1) The model parameter learning. It estimates the model parameters λ=(A,B,π) in the case of a known alert description sequence *O* such that the alert description sequence probability $P\left(O|\lambda \right)$ is maximized under the model. (2) Decoding. Given the model λ=(A,B,π) and the alert description sequence *O*, it finds the maximum state sequence corresponding to the conditional probability of the given sequence.

### 3.2. Multi-Step Attack Detection Process

**Figure 1:**
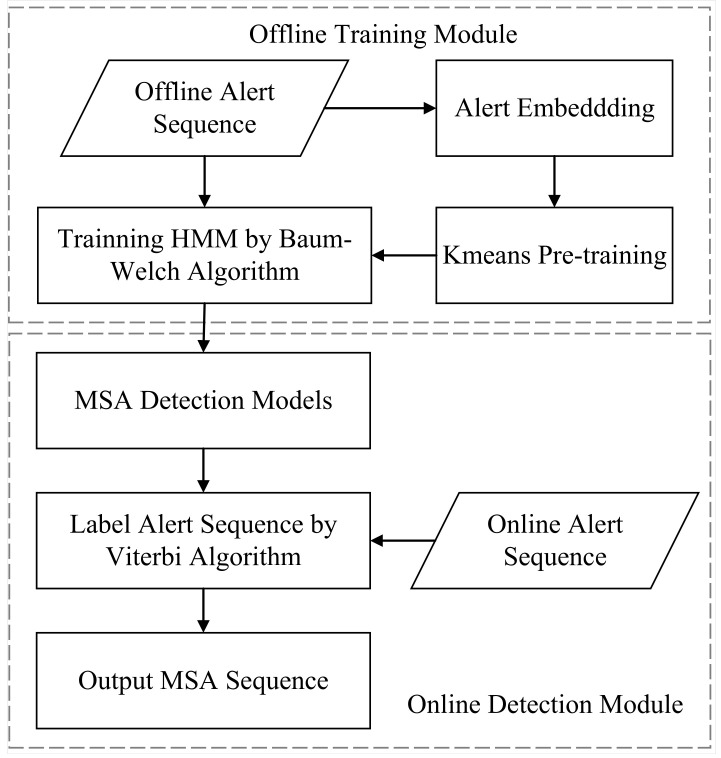
The proposed MSA-detection flowchart.

This paper proposes the following MSA detection process based on the above HMM, as shown in Figure 1. The process contains two parts: offline training and online detection.

The offline training includes alert embedding, K-means pre-training, Baum–Welch training, etc. As HMM cannot directly process the alert description in each piece of alert data, the alert description is preprocessed through the alert embedding and embedded into the digital vector so that the alert description with similar semantics in the same attack phase can be mapped to the vector space in a similar location.

The K-means algorithm pre-trains the alert description transfer probability matrix *B* in the HMM parameters to provide an unsupervised training phase with information on the semantics of the alert description and the order of the attack phases. The unsupervised training iteratively updates the HMM parameters with the Baum–Welch algorithm and finally outputs the HMM parameters. Online detection uses the Viterbi algorithm to find the sequence of attack steps *Q* that best matches the online alert sequence *O*. The main components are described in detail below.

#### 3.2.1. Alert Embedding

To solve one-hot encoding that cannot capture the relationship between the alert description and extract the semantic knowledge in the alert description, we employ the Word2Vec model to convert the alert description into continuous values of low dimensionality and map the semantically similar alert description to similar locations in the vector space. The Word2vec includes two training methods, CBOW and Skip-gram. Since the word vectors obtained by the Skip-gram method are more accurate when the amount of data contained in the alert description is small [24], the Skip-gram approach is chosen to train the word embedding model in this paper.

**Figure 2:**
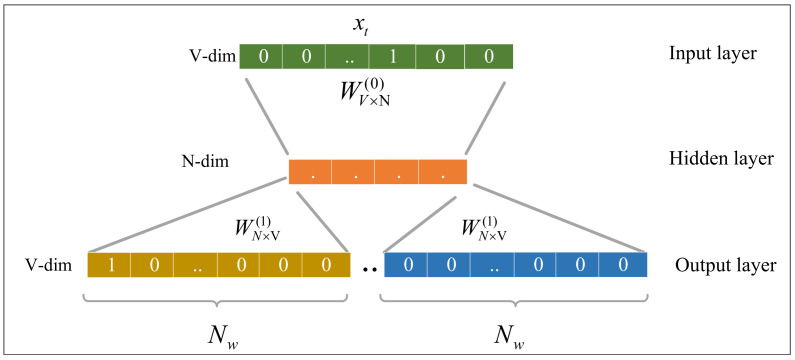
Skip-gram model architecture.

The Skip-gram architecture is shown in Figure 2, where the model’s input is vector *x* represented by a one-hot encoding. The *t*-th row of the weight matrix $W^{\left(0\right)}$ represents the weight of the *t*-th word in the vocabulary. Each word vector in the model has an N×V dimensional output vector $W^{\left(1\right)}$. The hidden layer contains *N* nodes, denoted by the symbol $N_{d}$. The hidden layer input is the weighted sum of the input layer. The output layer is $N_{w}$ windows of the current word *x*. The specific flow of the alert description embedding model is as follows.

<1> First, the alert description *V* contained in the IDS rules is segmented into multiple words. The list of words contained in all rule texts is the system’s vocabulary.
<2> Then, all words in the vocabulary are one-hot encoded and fed into the model to predict the $N_{w}$ windows of the current word *x* and update the hidden layer weights through backpropagation.

When the training is completed, the embedded word vector of a word can be obtained by its one-hot encoding.

The processing flow for converting alert descriptions to alert vectors is as follows.

<1> Segments the alert description to obtain all the words contained in the alert description.
<2> Uses the Word2Vec model to obtain the code corresponding to each word of the alert description and obtain the dimensional word vector corresponding to the alert description.
<3> Obtains the embedded vector of the alert description by computing the average value of all word vectors in the alert description.

**Figure 3:**
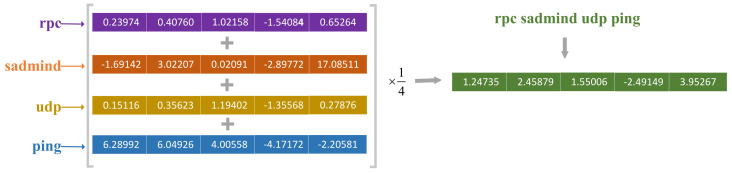
An example of an alert vector.

To facilitate understanding, we give an example of converting alert description to alert vectors in Figure 3. The alert description is “rpc sadmind udp ping”. After segmentation, we obtain four words “rpc”, “sadmind”, “udp”, and “ping”. The dimension of the embedding vector was set as five-dimensional. According to the Word2Vec model, we obtain four five-dimensional word embedding vectors. We can obtain the alert embedding vector for the alert description by taking the mean value of the sum of these four five-dimensional vectors.

#### 3.2.2. K-Means Pre-Training

To pre-train the transfer probability matrix *B* of the alert description, we cluster the alert description with the K-means algorithm. This algorithm first clusters the alert description with similar semantics into the same group. Then, it marks the attack phase according to the earliest alerts in each cluster and calculates the transfer probability of the alert vector based on the distance from the alert vector to each cluster center. The K-means algorithm occupies O((m+K)∗n) memory and O(tmnK) time, where *t* denotes the number of iterations, *m* denotes the number of data, *n* denotes the dimension of features, and *k* denotes the number of clusters. The specific steps are as follows:

<1> Initialization. Randomly selects *N* initial centers of mass, where *N* denotes the existence of *N* phases in an MSA.
<2> Alert de-duplication. First, we sort the alerts according to the time of occurrence and then check if there are alerts in the set with the same text as the current alert. If it does not exist, the alert is added to the set. After iterating through all alerts, we can obtain the set of non-redundant alerts.
<3> Alert embedding. We convert the alert description in the alerts set into $N_{d}$ alert vectors with the alert embedding model.
<4> Alert Clustering.
  (a) Traverses all $N_{d}$ dimensional alerts and assigns the alerts embedding vector to the nearest center of mass based on the distance between the alert and the center of mass. The distance between the alert vector *x* and the center of mass *y* is measured using the Euclidean distance, calculated as in Equation (18). After all alert embedding vectors are assigned, the center of mass $C_{i}$ is updated by Equation (19).
    (18) $$dist\left(x,y\right)=\sqrt{\sum_{i=1}^{n}{\left(x_{i}-y_{i}\right)}^{2}}$$
    (19) $$C_{i}=\frac{1}{\left|C_{i}\right|}\sum_{v\in C_{i}}v$$
  (b) Repeats step 4a until the center of mass no longer changes. The alert description of the same attack phase is divided into the same attack phase cluster. Then, goes to step <5> of Section 3.2.2.
<5> Attack phase determination. After step <5> of Section 3.2.2, each cluster contains a certain number of alerts. We determine the attack phase each cluster belongs to based on the earliest alert in each cluster.
<6> The alert description center-of-mass distance matrix *R* calculation. This matrix represents the inverse distance from the *M* alert vectors to the *N* attack phase cluster centers, calculated in Equations (20) and (21).
  (20) $$r_{i}\left(j\right)=\frac{1}{dist(v_{i},C_{j})}$$
  (21) $$R_{M\times N}=\left[\begin{array}{ccc} r_{1}\left(1\right) & \cdots & r_{1}\left(N\right)\\ \vdots & \ddots & \vdots \\ r_{M}\left(1\right) & \cdots & r_{M}\left(N\right) \end{array}\right]$$
<7> Alert description transfer probability matrix *B* calculation. We first transpose the matrix *R* of the distance between the alert description and the center of mass by Equation (22). We convert the distance into the probability of generating the alert description in the MSA phase using Equation (23). With the above steps, the alert description transfer probability matrix *B* is obtained.
  (22) $$R_{M\times N}^{T}\left[\begin{array}{ccc} r_{1}\left(1\right) & \cdots & r_{1}\left(M\right)\\ \vdots & \ddots & \vdots \\ r_{N}\left(1\right) & \cdots & r_{N}\left(M\right) \end{array}\right]$$
  (23) $$B=\left[\begin{array}{ccc} \frac{r_{1}\left(1\right)}{\sum_{i=1}^{M}r_{1}\left(i\right)} & \cdots & \frac{r_{1}\left(M\right)}{\sum_{i=1}^{M}r_{1}\left(i\right)}\\ \vdots & \ddots & \vdots \\ \frac{r_{N}\left(1\right)}{\sum_{i=1}^{M}r_{N}\left(i\right)} & \cdots & \frac{r_{N}\left(M\right)}{\sum_{i=1}^{M}r_{N}\left(i\right)} \end{array}\right]$$

#### 3.2.3. Unsupervised Training

We used the Baum–Welch algorithm to train further the triplet λ=<A,B,π> to obtain the final model parameters. The Baum–Welch algorithm finds the maximum likelihood estimates of the parameters in the HMM by iteratively computing the parameters using the forward-backward algorithm during the iterative process. The forward algorithm is shown in Equations (24) and (25). It is used to calculate the probability that the state $q_{t}$ is *j* when the sequence is observed from moment 1 to moment *t*, given the parameter λ. The backward algorithm is shown in Equations (26) and (27). It calculates the probability of observing the sequence from *t* to the *T* given the parameter λ and the *t*-th state $q_{t}$ as *i*.

(24) $$\alpha_{t}\left(j\right)=p\left(o_{1},o_{2},\ldots ,o_{t},q_{t}=j|\lambda \right)$$

(25) $$\alpha_{t}\left(j\right)=\sum_{i=1}^{N}\alpha_{t-1}\left(i\right)a_{ij}b_{j}{(o}_{t})$$

(26) $$\beta_{t}\left(i\right)=p\left(o_{t+1,}o_{t+2,}...,o_{T}|q_{t}=i,\lambda \right)$$

(27) $$\beta_{t}\left(i\right)=\sum_{j=1}^{N}a_{ij}b_{j}\left(o_{t+1}\right)\beta_{t+1}\left(j\right)$$

The Baum–Welch algorithm first initializes parameter λ=<A,B,π> and then repeats the expectation and maximization steps. The above K-means pre-training step initializes the alert description transition probability matrix B and the initial values. The Baum–Welch algorithm occupies memory and time are $O(K^{2}N)$, where *K* denotes the number of states and *N* denotes the number of time steps. The algorithm detail as follows.

<1> The initial attack phase probability matrix π setting up. Since the alert sequence is divided by the same interval or length, the initial probability expectation for each attack phase in a multi-step attack is 1/N.
<2> The attack phase transition matrix *A*. The attack phase transition matrix *A* is usually initialized by 1/N. The initialization is shown in Equation (28).
  (28) $$a_{ij}=\frac{1}{N},1\leq i\leq N,1\leq j\leq N$$
<3> Expectation step. Calculates the probability $\gamma_{t}\left(j\right)$ of the attack phase *j* of time *t*, and the expectation $\xi_{t}\left(i,j\right)$ of attack phase *i* at time *t* converted to attack phase *j* at time t+1. The calculations are as shown in Equations (29) and (30).
  (29) $$y_{t}\left(j\right)=\frac{\alpha_{t}\left(j\right)\beta_{t}\left(j\right)}{\sum_{j=1}^{N}\alpha_{t}\left(j\right)\beta_{t}\left(j\right)}$$
  (30) $$\xi_{t}\left(i,j\right)=\frac{\alpha_{t}\left(i\right)a_{ij}b_{j}\left(o_{t+1}\right)\beta_{t+1}\left(j\right)}{\sum_{j=1}^{N}\alpha_{t}\left(j\right)\beta_{t}\left(j\right)}$$
<4> Maximization step. We used $\gamma_{t}\left(j\right),\xi_{t}\left(i,j\right)$ to recalculate the new matrix A,B with Equations (31) and (32).
  (31) $${\hat{a}}_{i,j}=\frac{\sum_{t=1}^{T-1}\xi_{t}\left(i,j\right)}{\sum_{t=1}^{T-1}\sum_{k=1}^{N}\xi_{t}\left(i,k\right)}$$
  (32) $${\hat{b}}_{j}\left(v_{k}\right)=\frac{\sum_{t=1,s.t.o_{t}=v_{k}}^{T-1}y_{t}\left(j\right)}{\sum_{t=1}^{T-1}y_{t}\left(j\right)}$$
<5> Repeat steps <3> of Section 3.2.3 and steps <4> of Section 3.2.3 until the model converges and then steps <6> of Section 3.2.3.
<6> Output MSA detection model parameters λ=<A,B,π>.

#### 3.2.4. On-Line Detection

The online detection infers the attack phase sequence $Q_{T}=\left\{q_{1},q_{2},\ldots ,q_{T}\right\}$ based on the alert sequence $O=\{o_{1},o_{2},\ldots ,o_{T}\}$ and the multi-step attack detection model λ=<A,B,π>. This process is the decoding process of HMM. The main decoding algorithm is the Viterbi algorithm. Similar to the forward algorithm, the Viterbi algorithm is also a dynamic programming algorithm. It computes the most probable path to the current state recursively by using backward pointers, as shown in Equation (33).

The recursive formula for $v_{t}\left(j\right)$ is shown in Equation (34), where $v_{t}\left(j\right)$ represents the maximum probability of occurrence of the attack phase sequence $Q_{t-1}=\left\{q_{1},q_{2},\ldots ,q_{t-1}\right\}$ for a known alert sequence *O* with state $q_{t}$ given parameter λ. The equation for solving the multi-step attack phase sequence *Q* corresponding to the alert sequence *O* is shown in Equation (35). The Viterbi algorithm occupies O(KN) memory and $O(K^{2}N)$ time, where *K* denotes the number of states and *N* denotes the number of time steps.

(33) $$v_{t}\left(j\right)=\max_{q_{1},\ldots ,q_{t-1}}p\left(q_{1},\ldots ,q_{t-1},o_{1},\ldots ,o_{t},q_{t}=j|\lambda \right),1<=j<=N,1<=t<=T$$

(34) $$v_{t}j=\max_{i}v_{t-1}\left(i\right)a_{ij}b_{j}\left(o_{t}\right),1<=i,j<=N,1<t<=T$$

(35) $$Q_{T}=\arg\max_{i}v_{T}\left(i\right),1<=i<=N$$

## 4. Experiments and Results Analysis

In this section, we detail comparative experiments to evaluate the proposed method. The original Baum–Welch algorithm [18,19] is the most-used unsupervised learning algorithm in HMM-based multi-step attack models. Therefore, this training method was chosen as the benchmark algorithm to validate the effectiveness of our proposed method. In addition, Larue [25] used the K-means method to solve the initialization point-selection problem.

First, a large number of state-accurate parameters are constructed to descript the observation sequences, and then we used the K-means algorithm to reduce the number of states and maintain the accuracy of the HMM parameters. Chadza [26] proposed a transfer learning (TL)-method to transfer knowledge learned from a labeled source dataset to a new, unlabeled target dataset to optimize the MSA model parameters. In this paper, the TL-based Different Evolution (DE) algorithm has the best detection effect on the overall sequence decoding. Thus, we also compare our method with these methods.

The main difference between the algorithm proposed in this paper and the selected benchmark methods is the way the model parameters are initialized. Therefore, we first initialized the HMM with the initial values obtained by three different initialization methods, then used the training set to train the detection model, and finally, the effectiveness of the proposed method was verified by comparing the detection effect of the detection model on the test set. We used the labels of each attack stage as positive samples separately and the labels of other attack stages as negative samples. TP is the number of positive samples correctly labeled as positive, TN is the number of negative samples correctly labeled as negative, FP is the number of negative samples labeled as positive, and FN is the number of positive samples labeled as negative.

The accuracy, precision, recall, and F1 score are classical evaluation criteria within the field of multi-step attacks detection. To verify that the proposed training method obtains the detection effect of the model, we used the average of precision, recall, and F1 scores for each attack phase, shown in Equations (36)–(38) and accuracy to evaluate shown in Equation (39).

(36) $$Precision=\frac{TP}{TP+FP}$$

(37) $$Recall=\frac{TP}{TP+FN}$$

(38) $$F1=\frac{2*Precision*Recall}{Precision+Recall}$$

(39) $$Accuracy=\frac{number\;of\;correct\;predictions}{total\;predictions}$$

This section is organized as follows. Section 4.1 presents the implementation details of the pre-training method. Section 4.2 describes the dataset for evaluating the performance of the model. Section 4.3 shows the experimental setup. Section 4.4 shows the results and discusses.

### 4.1. Implementation

In the alert embedding part, this paper uses deeplearning4j to train the alert embedding model in the java environment (jdk version 1.8). First, instantiate the SentenceIterator class to iteratively access the alert description during training. Then, instantiate the TokenizerFactory class for tokenizing the alert description during the training process. Then, enter the alert description and the two classes instantiated in the above process as parameters into the Word2Vec class and instantiate the Word2Vec class. Finally, call the fit() method of the Word2Vec class to train the model.

In the K-means pre-training part, this paper uses the *K-means* API in deeplearning4j to pre-train the multi-step attack model parameter. First, call the setup() method of the KMeansClustering class to instantiate the cluster. Then, call the getWordVector() method of the Word2Vec class to convert the alert descriptions into an alert vector. Alert vector is input as a parameter to the applyTo() method of the KMeansClustering class to cluster the alert descriptions. When the clustering is complete, we convert the distance of the alert vector to the cluster centroid into the alert description transition probability matrix *B* using the Equations (20)–(23).

In the model training part, this paper uses jahmm0.6.1 to train the HMM. We call the Hmm.setOpdf() method to initialize the jahmm.Opdf object with the alert description transition probability matrix *B* obtained by pre-training. Then, the alert text sequence O is input as a parameter to the BaumWelchLearner.learn() method for learning to generate a multi-step attack detection model.

### 4.2. Dataset

This paper evaluates the efficiency of the proposed MSA detection model using the DARPA 2000 public dataset [27], the DEFCON 21 CTF public dataset [28] and the ISCXIDS 2012 public dataset [29]. The DARPA2000 evaluation dataset covers a wide range of attack methods and is the most comprehensive and up-to-date MSA dataset in the DARPA dataset. This dataset is a generally accepted and widely used benchmark dataset in the field of MSA research. The DEFCON21 CTF data set is the captured data packets of the 21st DEFCON CTF conference offline competition, which contains multiple real attack steps.

We used snort to detect abnormal traffic in the data packets to generate alerts, and then used the alerts to reconstruct multiple-step attack scenarios. The ISCXIDS 2012 dataset is the latest MSA dataset published by the Canadian Institute for Cybersecurity Research. This dataset consists of real networks and traffic and contains a complete multi-step attack scenario, which contains more attack types compared to the DARPA 2000 dataset and DEFCON21 CTF dataset. Thus, we chose these datasets to evaluate the efficiency of the model.

The DARPA 2000 dataset contains two attack sequences, LLDOS 1.0 and LLDOS 2.0.2. As the attack sequence LLDOS 2.0.2 generates fewer alert data, we used LLDOS 1.0 to validate the model. We used LLDOS 1.0 to verify the model’s effectiveness. The LLDOS 1.0 attack sequence contains five attack phases: (1) IP scan. This attack phase is used to discover surviving hosts. (2) The adversary probe. This phase confirms whether the sadmind service is running on the host. (3) Breakins. In this phase, the attacker exploits a vulnerability in the sadmind software on Solaris. The sadmind software vulnerability to obtain root privileges of the host. (4) Installation and initialization. This phase installs and initializes the trojan mstream DDoS software. (5) Launch attack. In this phase, the attacker launches a DDOS attack on the specified IP.

We reconstruct a multi-step attack sequence in the DEFCON21 CTF dataset by concatenating single but dependent attack steps in order. The reconstructed multi-step attack consists of three attack steps: (1) Port scanning, the attack phase scans the host’s open ports to identify vulnerable services. (2) POODLE exploit, the attack phase rolls back the SSLv3 encryption algorithm to perform a man-in-the-middle attack. (3) Code execution, the attacker directly injects operating system commands or codes into the background server remotely.

The ISCXIDS 2012 dataset contains five attack phases in the testbed-13jun.pcap packet [30]: (1) Adobe Reader vulnerability exploitation. This attack phase exploits the stack overflow vulnerability in Adobe Reader parsing a PDF to execute malicious code and gain control of the host. (2) Intranet asset scanning. The 192.168.1.0/24 and 192.168.2.0/24 hosts and ports are scanned. (3) MS08-067 remote overflow vulnerability exploitation. The attacker uses the 192.168.1.112 default open SMB service port 445 to send a special RPC, execute malicious code, and gain control of the host. (4) Intranet asset scanning. The attacker scanned 192.168.5.0/24 hosts and ports. (5) SQL injection attack. The attacker does an SQL injection attack on 192.168.5.123.

The MSA detection in this paper uses alerts generated by the IDS as input, and the dataset is real attack traffic stored as pcap packets; thus, it is necessary to convert the real attack traffic into alert data. In this paper, we used the tcpreplay v4.3.2 tool to replay LLDOS 1.0 and ISCXIDS 2012 packets, respectively, and used snort v2.9.7.0 to detect these flows and generate alert data. As shown in Table 1, the Inside contains 75,423 network packets and generates 573 alert records, including 16 types of alert descriptions. The DMZ contains 36,542 network packets and generates 1117 alert records, including 14 types of alert descriptions. We used the Inside data set to train our MSA detection model and used the DMZ data set to test our model.

The pcap of the DEFCON21 CTF dataset generates 516 alert records, including 16 types of alert descriptions. Since the dataset does not distinguish the attack stage, we cannot count the data packets of each stage; therefore, it is represented by −. ISCXIDS 2012 testbed-13jun.pcap contains a total of 5,763,149 network packets, generates 205 alert records, and contains 29 types of alert descriptions.

### 4.3. Experimental Setup

Our experiment implemented all the programming work on 64-bit Windows 10 based OS. The computer comprises an Intel core i-5 processor@ 3.20 GHz, 16 GB RAM. The graphics card is Intel(R) HD Graphics 530. As mentioned in Section 3.2.1 and Section 3.2.2, there are three parameters to be set during the experiment. (1) The dimension of the word embedding $N_{d}$, which means that the word is embedded into the dimension of the vector space. If the embedding dimension of the word vector is too high, the relationship between words will be diluted.

If the dimension is too low, words cannot be distinguished. Generally, the embedding dimension of the word vector is set between 200 and 400. Due to the small amount of alert corpus data; therefore, we chose the embedding dimension $N_{d}=200$. (2) The size of the window $N_{w}$, which represents the maximum range of the context predicted by the central word in the window. The window size is related to the sentence length in the corpus, and thus we counted the distribution of snort alert description sentence lengths and found that the average length of the alert description sentence was approximately five words; thus, we set $N_{w}=5$. (3) The number of clusters $N_{s}$, which represents the number of stages included in each multi-step attack scenario. In this experiment, the multi-step attack scenarios in DARPA 2000 dataset and ISCXIDS 2012 dataset consist of five steps, and thus we set the number of clusters to $N_{s}=5$. Whereas the DEFCON21 dataset consists of three steps; therefore, the number of clusters was set to $N_{s}=3$ in this dataset.

### 4.4. Results and Discussion

**Figure 4:**
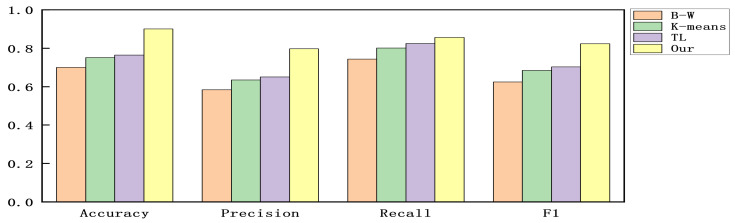
The results for DARPA 2000.

**Figure 5:**
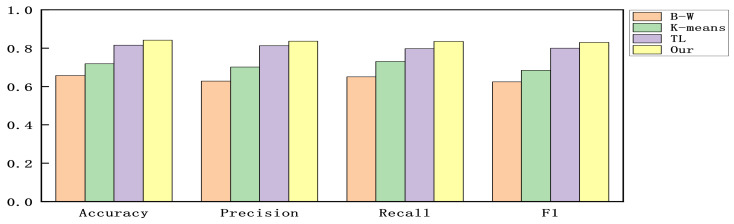
The results for DEFCON21.

**Figure 6:**
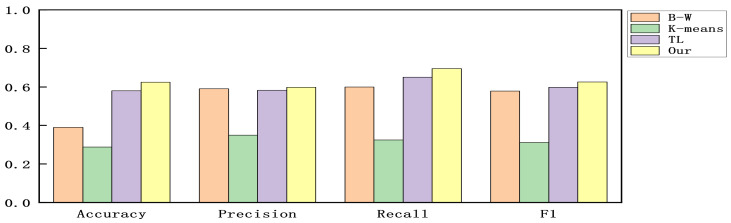
The results for ISCX2012.

We constructed experiments with the accuracy, precision, recall, and F1-score as evaluation criteria to verify the detection ability of the proposed model. Since all alerts are part of the multi-step attacks without benign alerts. To make the experimental results clear, we calculated the results by considering each attack phase as a positive sample and the other attack phases as negative samples and then used the mean value of the results of each attack phase to indicate the detection effectiveness of the model in the dataset. The results are shown in Figure 4, Figure 5 and Figure 6.

As can be seen in Figure 4, Figure 5 and Figure 6. Compared with the original Baum–Welch algorithm [18,19] and K-means based algorithm [25], the detection effect of the model obtained by our proposed training method is significantly improved. Compared with the TL-based DE algorithm [26], the model obtained by our proposed training method has better detection performance and does not depend on external label data. In the method proposed in this paper, since alerts at the same stage have semantic similarity, the pre-training part delineates the attack stage to which each alert belongs in advance by clustering the semantic information of the alerts.

Then, we convert the distance of the alert vector to each attack stage into the probability of generating an alert in each attack stage instead of the initial value of Baum–Welch, thus, avoiding the problem of the model falling into a local optimum. Compared with the other three training methods, this method incorporates semantic information in the model training and optimizes the initial parameters of the model, and thus the detection effect is improved.

**Figure 7:**
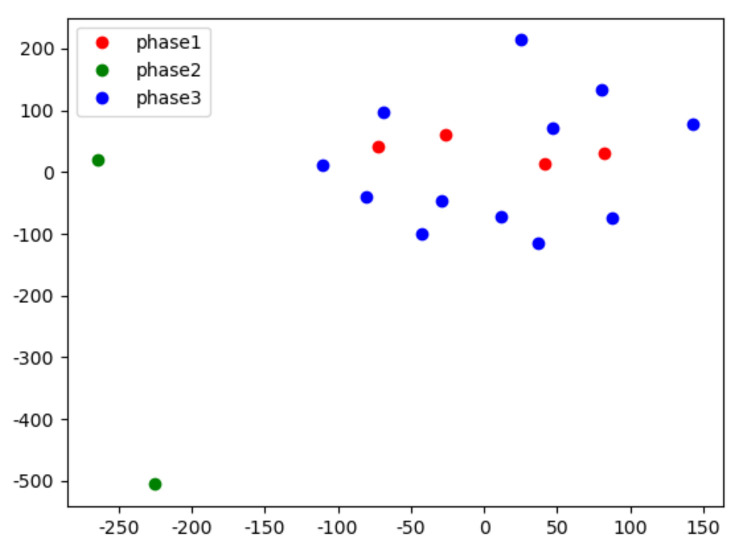
The ISCXIDS 2012 partial alert vector.

Compared with the experimental results on the LLDOS 1.0 dataset and DEFCON21 dataset, the detection rate of the proposed method on the ISCXIDS 2012 dataset is significantly lower. The reasons are as follows: As shown in Figure 7, the ISCXIDS 2012 dataset has more alert description categories, while the alert description still contains essentially similar words.

This results in a higher repetition rate of words contained in the alert description indifferent attack phases in the ISCXIDS2012 dataset. In the parameter pre-training phase, the alerts are closer to the inter-cluster boundaries, and the difference in distance from the centers of each cluster is smaller. This increases the difference between initialized and actual alert description transfer probability matrices during the pre-training process and eventually affects the detection effectiveness of the MSA model.

Elements of the initial parameter matrix trained by each training method are shown in Table 2. The effectiveness of our proposed method is confirmed by comparing the initial parameters with the other three training methods. In the real attack scenario, the $s_{1}$ attack phase only generates $v_{1}$, $v_{2}$, and $v_{3}$ alerts, the $s_{2}$ attack phase only generates $v_{4}$ and $v_{5}$ alerts, and the $s_{5}$ attack phase only generates $v_{16}$. In the initial value based on K-means training, the probability of generating $v_{2}$ and $v_{3}$ in the attack phase $s_{1}$ is 0, and the parameters of the model are different from the attack scenario.

Therefore, the detection effect of the multi-step attack model trained by this method is lower. TL-based DE and Baum–Welch initialize the model parameters using the average initialization method. This initial value does not provide gain to the model, and thus the original Baum–Welch algorithm is less effective compared to the models obtained by other algorithms. The TL-based DE model training method also uses a uniform initial value; however, it uses a transfer learning algorithm to optimize the model parameters and improve the detection of the model.

Compared to the initial parameters of the model described above, the initial parameters of the model trained with our proposed method are closer to the optimal solution of the model. The training method proposed in this paper uses the semantic similarity of $v_{1}$, $v_{2}$, and $v_{3}$ to pre-classify them successfully into the $s_{1}$ attack phase, and thus it can be seen from the table that the highest probability values of $v_{1}$, $v_{2}$, and $v_{3}$ alerts are generated by $s_{1}$. Similarly, we successfully pre-assign the alerts generated by other attack phases to the attack phase to which they belong.

The second attack phase is scanning the sadmind service, while the third attack phase is exploiting the sadmind vulnerability; thereby, the semantics of the alerts generated by the third attack phase are slightly similar to those generated by the second attack phase, and we can see that $s_{3}$ generates $v_{4}$, $v_{5}$ with higher probability. In addition, the method proposed in this paper converts the distances between alerts and each attack phase into matrix elements.

Therefore, it can be seen from the table that most of the elements are non-zero. Most importantly, the initial parameters obtained by the pre-training method proposed in this paper are closer to the real multi-step attack than the other three training methods. Therefore, pre-training the parametric model using this method can avoid falling into a local optimum.

### 4.5. Detection Performance

In our experiment, we took the interval between the online detection start time and end time as the delay. In addition, the memory consumed in the online detection was calculated by two functions: runtime.totalMemory,runtime.freeMemory(). The results are shown in Table 3.

Compared with the other three methods, this model trained by our method has less delay for online detection. The memory consumption of our model is the same as the B-W-based method and K-means-based method but less than the TL-DE-based method. Compared to the other two datasets, the ISCX2012 dataset contains more alert descriptions, and the process of labeling the alert sequence is more complicated. Therefore, the model takes more time to process this alert sequence.

As above mentioned, our proposed model can process the alert sequence in milliseconds. As the multi-step attack data is sparse, our proposed online detection method is sufficient to complete the labeling task in real-time.

## 5. Conclusions

This paper solved the problem where the current Baum–Welch initialization method easily causes the model to fall into a locally optimal solution. Based on the idea that the alerts generated in the same attack stage have high semantic similarity, this paper used semantic clustering to aggregate the alerts belonging to the same attack stage. Then, we converted the distance of the alert vector for each attack stage to the probability of generating an alert for each attack stage and replaced the initial value of Baum–Welch with the initial value optimized by semantic knowledge to avoid the model becoming stuck in a locally optimal solution. We then evaluated the proposed model using the DARPA 2000, DEFCON21 CTF, and ISCXIDS 2012 datasets.

The results show that the initial values of HMM obtained by the initial value training method proposed in this paper are more consistent with the actual attack scenario compared with the original Baum–Welch method, the K-means-based method, and the TL DE-based method. The initial parameters are closer to the optimal parameters of the model, and training the model with such initial values can prevent the model from falling into local optimal solutions.

Thus, the detection effect was better than the other three training methods. However, the detection rate of MSA decreased significantly when the alert described more categories. Therefore, in future work, we plan to train the HMM model with a semi-supervised algorithm to improve the detection effect of the model on a large dataset of alert description categories.

## Figures and Tables

**Table 1:** The datasets of the multi-step attack sequence.

Dataset	Data Source	Attack Phase	Data Packages	Alert Description
LLDOS 1.0	Inside	$s_{1}$	40	40
		$s_{2}$	158	151
		$s_{3}$	225	42
		$s_{4}$	520	74
		$s_{5}$	74,480	266
	DMZ	$s_{1}$	785	785
		$s_{2}$	148	135
		$s_{3}$	530	96
		$s_{4}$	526	100
		$s_{5}$	34,553	1
DEFCON21 CTF	-	$s_{1}$	-	89
		$s_{2}$	-	179
		$s_{3}$	-	248
ISCXIDS 2012	testbed-13jun	$s_{1}$	4,294,502	54
		$s_{2}$	27,928	117
		$s_{3}$	61,698	1
		$s_{4}$	37,240	23
		$s_{5}$	1,341,785	10

**Table 2:** The initial parameters of the alert description transition probability matrix *B* in the DARPA 2000 dataset. $s_{i}$ denotes the *i* attack phase, $v_{j}$ denotes the *j* alert description, and the element $b_{i}\left(j\right)$ in the matrix denotes the probability of the *i* attack phase generate an alert description *j*.

Attack Phase	Training Method	Alert Description						
		$v_{1}$	$v_{2}$	$v_{3}$	$v_{4}$	$v_{5}$	⋯	$v_{16}$
$s_{1}$	TL DE and B-W	0.0625	0.0625	0.0625	0.0625	0.0625	⋯	0.0625
	K-means	**1.0**	**0.00**	**0.00**	0.00	0.00	⋯	0.00
	Our	**0.30**	**0.29**	**0.20**	0.00	0.01	⋯	0.00
$s_{2}$	TL DE and B-W	0.0625	0.0625	0.0625	0.0625	0.0625	⋯	0.0625
	K-means	0.00	0.00	0.00	0.91	0.09	⋯	0.00
	Our	0.01	0.00	0.01	**0.32**	**0.45**	⋯	0.00
$s_{3}$	TL DE and B-W	0.0625	0.0625	0.0625	0.0625	0.0625	⋯	0.0625
	K-means	0.00	0.00	0.00	0.00	0.00	⋯	0.00
	Our	0.04	0.02	0.01	0.09	0.10	⋯	0.00
$s_{4}$	TL DE and B-W	0.0625	0.0625	0.0625	0.0625	0.0625	⋯	0.0625
	K-means	0.00	0.00	0.00	0.00	0.00	⋯	0.00
	Our	0.01	0.01	0.01	0.01	0.00	⋯	0.00
$s_{5}$	TL DE and B-W	0.0625	0.0625	0.0625	0.0625	0.0625	⋯	0.0625
	K-means	0.00	0.00	0.00	0.00	0.00	⋯	0.34
	Our	0.00	0.00	0.00	0.05	0.01	⋯	**0.65**

**Table 3:** Online detection delay and the number of alerts.

Dataset	Number of Alerts	Training Method	Delay (ms)	Memory Consumption (MB)
DARPA 2000	1117	B-W	6	12
		K-means	6	12
		TL	4.6	17
		Our	4	12
DEFCON21 CTF	516	B-W	4.5	12
		K-means	4.5	12
		TL	4.8	17
		Our	4.3	12
ISCX2012	205	B-W	4.5	12
		K-means	4.4	12
		TL	4.4	12
		Our	4.3	12

## Data Availability

Publicly available datasets were used in this study. This data can be found here: (https://www.ll.mit.edu/r-d/datasets/2000-darpa-intrusion-detection-scenario-specific-datasets [2000 DARPA INTRUSION DETECTION SCENARIO SPECIFIC DATASETS], https://www.unb.ca/cic/datasets/ids.html (accessed on 21 February 2022) (Intrusion detection evaluation dataset (ISCXIDS2012))).
